# A Mathematical Framework for Combining Decisions of Multiple Experts toward Accurate and Remote Diagnosis of Malaria Using Tele-Microscopy

**DOI:** 10.1371/journal.pone.0046192

**Published:** 2012-10-11

**Authors:** Sam Mavandadi, Steve Feng, Frank Yu, Stoyan Dimitrov, Karin Nielsen-Saines, William R. Prescott, Aydogan Ozcan

**Affiliations:** 1 Electrical Engineering Department, University of California Los Angeles, Los Angeles, California, United States of America; 2 Bioengineering Department, University of California Los Angeles, Los Angeles, California, United States of America; 3 Division of Infectious Diseases, Department of Pediatrics, School of Medicine, University of California Los Angeles, Los Angeles, California, United States of America; 4 Hydas World Health, Hershey, Pennsylvania, United States of America; 5 California NanoSystems Institute, University of California Los Angeles, Los Angeles, California, United States of America; 6 Department of Surgery, David Geffen School of Medicine, University of California Los Angeles, Los Angeles, California, United States of America; Centro de Pesquisa Rene Rachou/Fundação Oswaldo Cruz (Fiocruz-Minas), Brazil

## Abstract

We propose a methodology for digitally fusing diagnostic decisions made by multiple medical experts in order to improve accuracy of diagnosis. Toward this goal, we report an experimental study involving nine experts, where each one was given more than 8,000 digital microscopic images of individual human red blood cells and asked to identify malaria infected cells. The results of this experiment reveal that even highly trained medical experts are not always self-consistent in their diagnostic decisions and that there exists a fair level of disagreement among experts, even for binary decisions (i.e., infected vs. uninfected). To tackle this general medical diagnosis problem, we propose a probabilistic algorithm to fuse the decisions made by trained medical experts to robustly achieve higher levels of accuracy when compared to individual experts making such decisions. By modelling the decisions of experts as a three component mixture model and solving for the underlying parameters using the Expectation Maximisation algorithm, we demonstrate the efficacy of our approach which significantly improves the overall diagnostic accuracy of malaria infected cells. Additionally, we present a mathematical framework for performing ‘slide-level’ diagnosis by using individual ‘cell-level’ diagnosis data, shedding more light on the statistical rules that should govern the routine practice in examination of e.g., thin blood smear samples. This framework could be generalized for various other tele-pathology needs, and can be used by trained experts within an efficient tele-medicine platform.

## Introduction

Accurate diagnosis of medical images, regardless of their source, is in general a task that requires high levels of expertise typically gained through many years of training and experience. As such it is expected that there should exist varying levels of diagnostic accuracy among medical professionals depending on their individual training. One challenge which renders investigation of this issue difficult is the lack of direct and easy access to error-free analysis techniques, which makes the quantification of diagnostics errors of individual experts difficult. On top of this, an individual diagnostic decision (e.g., diagnosis of malaria through a blood smear) is often made through investigation of smaller pieces of images (e.g., individual red blood cells or smaller field-of-views that make up the microscope slide), which further help hide individual cell-level diagnostic errors of experts. In this work, we shed more light on this issue, and aim to combine the decisions of multiple experts to reduce diagnostic errors, and remotely monitor and compare performances of individual experts.

Multi-expert analysis and learning from multiple labels are areas of substantial research in machine learning [1]–[11]. Typically, a multi-expert system consists of multiple expert algorithms for some pattern recognition task and the overall system aims to optimally combine the decisions that are produced by these independent experts, with the fusion algorithm being a key component in the technique. The general idea is that the ‘combined’ performance of all the experts is better than any single one. Multi-label learning systems attempt to learn and identify the correct labels from a multitude of available labels that may have been generated by completely independent sources. Though in the Machine Learning literature an expert is normally taken to be an instance of a classification algorithm, in this work we will use the term ‘expert’ to refer to an *expert medical professional*, who ‘effectively’ acts as an independent classifier.

Computerised analysis of medical images is also an area that has experienced rapid advancements over the past decade [12]–[21]. Furthermore, statistical learning approaches are becoming more and more prevalent in both generating automated decisions and combining the decisions made by human experts [13], [14]. To give some examples, Warfield et al. [14] describe a methodology for fusing image segmentations made by multiple experts by simultaneously generating an estimate for the true segmentation and performance of the/emphsegmentors; and in a study by Raykar et al. [13] a multi-expert approach to the analysis of mammograms is described. Additionally, there is a large body of work in the area of multi-reader analysis in the medical imaging literature. The majority of these studies, however, are aimed at analysing the reader performances and agreement for specific tasks [22]–[28].

In this work, we propose a multi-label learning technique for combining diagnostic decisions generated by a set of independent medical experts (e.g., pathologists) for identifying human red blood cells (RBCs) that are infected by malaria. We chose malaria as our case-study since it is a disease that still afflicts a large number of people around the globe, and is most prevalent in impoverished and remote locations of the world, making it a good candidate for remote-diagnosis methodologies. According to the World Health Organization (WHO), there were an estimated 174 million cases of malaria in 2010 resulting in 655,000 deaths [29]. Laboratory diagnosis of malaria relies on traditional optical microscopy—one of the gold standard methods for detecting the parasite—in the vast majority of cases. The number of patients tested through this type of examination reached an estimated total of 165 million in 2010 [29], with the majority of cases coming from India.

The methodology proposed in this work can be adapted into a crowd-sourcing platform for remote diagnosis. Along these lines, we have recently shown that through entertaining digital games, it is possible to combine the binary decisions of minimally trained non-expert individuals to identify human RBCs infected with malaria [30]. In this earlier work, we used control images with ‘known’ labels to estimate the statistical behaviour of decisions made by individual gamers, which was then used to combine all the gamers' responses through a *Maximum a posteriori Probability* (MAP) estimation, achieving highly accurate overall decisions (coming close to the diagnoses made by a medical expert). In this current work however, we address another important diagnostic problem where the gold standard performance metrics are missing; i.e., we **do not** have access to any labelled data. Therefore, we approach the problem of labelling RBCs that are potentially infected with malaria parasites, by looking at the decisions that are made by a group of trained medical experts. We motivate this work by experimentally showing the level of discrepancy that exists among nine different experts, as well as the self-inconsistency that exists in the responses of each individual expert. We demonstrate that by using the Expectation Maximisation (EM) algorithm [31], we can combine the decisions made by such experts to generate more reliable diagnostic decisions at the single cell level.

We also present a mathematical framework for converting these individual ‘cell-level’ diagnosis results to ‘slide-level’ diagnosis, shedding more light on the statistical rules that should govern the routine practice in diagnosis and monitoring of malaria infected patients using e.g., thin blood smear samples.

We believe that the presented mathematical framework and the underlying digital infrastructure could be generalized for various other tele-medicine applications, toward creation of a cost-effective, efficient and accurate remote diagnostics platform.

## Methods

### Setup

**Figure 1:**
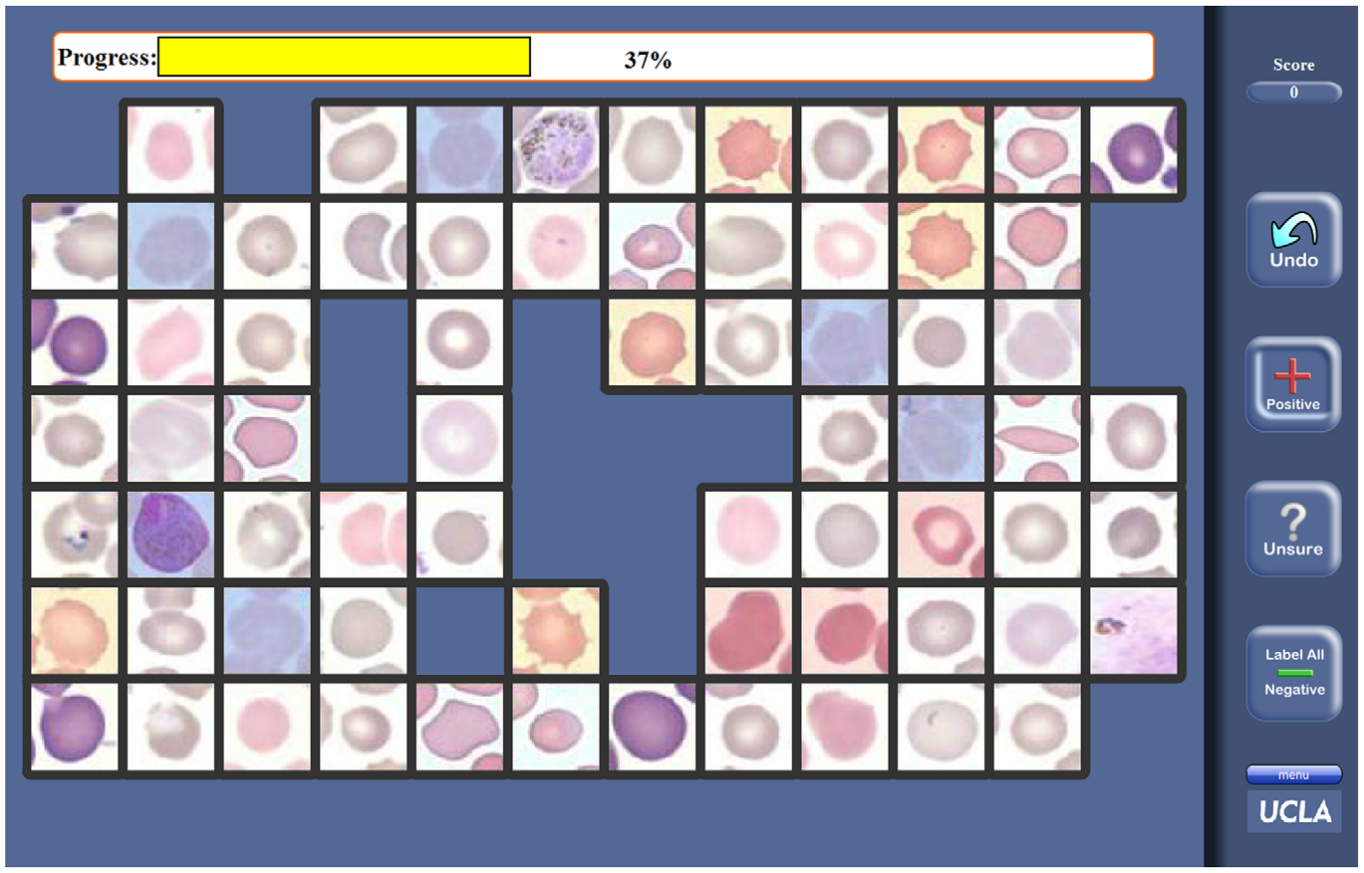
The browser-based interface for remote cell labelling. Each expert is allowed to navigate through the database of cell images, eliminating the infected cells and marking those that are questionable (i.e., cannot be reliably labelled as infected or uninfected).

In this work, we utilized 8,644 RBC images that were digitally cropped from Giemsa stained thin blood smears acquired from U.S. Centers for Disease Control and Prevention (CDC) database. This dataset of 8,664 images was derived from an original set of 2,888 images; i.e., each original image was rotated at multiples of 90 and randomly distributed within the final dataset. These images were originally captured using different digital microscopes through 100× objective lenses (with at least a numerical aperture of 1.0), and were digitized at 24 bits. These images were then remotely presented to each individual expert through a browser-based web interface as shown in Figure 1. This interface consists of multiple image *frames*, each containing a grid of individual RBC images. The size of the grid depends on the screen resolution of the computer accessing the interface and is automatically adjusted. The expert is asked to remove the infected and questionable (e.g., poor image quality, difficult to diagnose, etc.) cell images using the appropriate tools selectable from the side bar. Once all such images have been labelled, the remaining cells can all be labelled as *uninfected or healthy* using a *Label All Negative* button on the side bar. The experts are asked to log-in prior to starting the diagnosis, and their individual responses are recorded on our servers as they progress through the database of images. Note also that the experts were allowed to view and diagnose the images in multiple sessions and were not given any time constraints for completing the diagnosis task. All the slide readers were expert malaria diagnosticians and had clinical experience with reading of thin smears. In addition to these, we did not have any control on, nor did we enforce any conditions on the viewing devices of the observers. Any inconsistencies in the quality of their viewing hardware and conditions would be reflected in their diagnosis accuracies. For our mathematical framework, every expert is a statistical decision unit, and all the possible sources of error for an individual expert (e.g., relatively weaker training, poor eye sight, low display resolution, etc.) are treated as a lumped entity; and we do ‘not’ aim to investigate different factors that make up the overall error probability of an individual expert. Instead, one of the main goals of this work was to demonstrate that a group of experts could be digitally combined to significantly boost the accuracy of the final diagnostic decision, when compared to even the best individual of the group.

In the general scenario under consideration, we assume there are a total of N+1 medical images waiting to be diagnosed by M+1 experts. We also assume that the diagnosis is of a binary nature, meaning that it is either positive or negative, as in the case of malaria diagnosis. However, we also allow for the possibility that a particular image is of low quality preventing in some cases reliable diagnosis. As a result, each image can be labelled as *positive, negative,* or *questionable*.

If we had access to an infinite number of expert responses, and assuming that each expert produces the correct image label with more than 50% probability, then taking a simple majority vote for each image would produce the correct labels. This however is not the case practically, and we only have a limited number of experts available. Furthermore, if the error probabilities of the individual experts were known, then a MAP formulation [30] would be possible in order to find the most plausible labels for the original data. Therefore, in approaching this diagnosis problem, we need to simultaneously learn the image labels *and* the error probabilities associated with each expert, while maximising the posterior probability of the observed labels. To achieve this we assume a three-category mixture model for the original data, and use an EM algorithm to generate the Maximum Likelihood labels for the unknown cell images.

### Mixture Model Formulation

We assume that each image 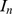 has one of three possible labels from the set 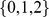 corresponding to the diagnostic decisions: *negative*, *positive*, and *questionable* images respectively. Therefore each input image belongs to one of three possible distributions corresponding to the three possible labels. This gives us a mixture model with 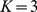 components.

**Figure 2:**
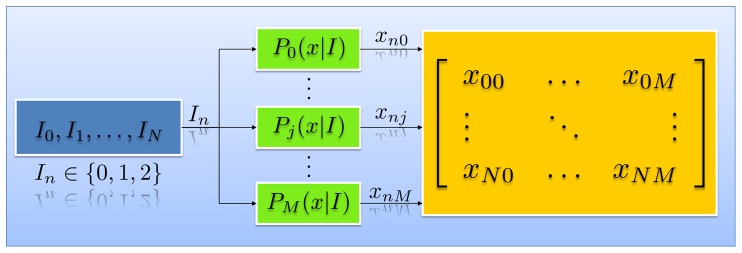
Forward model of the proposed setup. There are a total of 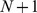 images with possible labels from 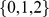 being sent to 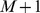 experts. The 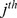 expert labels each image with a certain probability 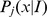. The final dataset consists of an 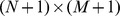 matrix of values from the set 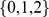.

**Figure 3:**
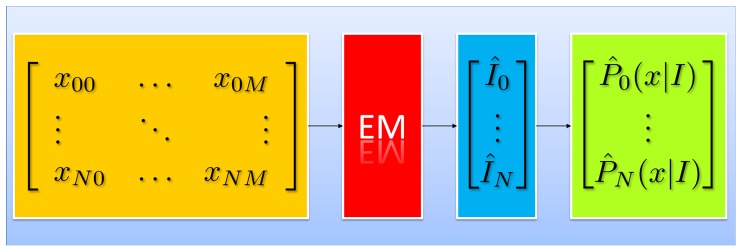
Decoding model of the proposed setup. The expert responses 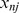 are treated as the observed variables and the true image labels 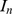 as the latent variables in a mixture model with parameters 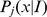. Expectation Maximisation (EM) is used to obtain the Maximum Likelihood solution to the data.

For each component 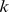, we assume the most general decision model for each user with six parameters describing the probability of the user's responses given the true labels of the images. Furthermore, we assume a 1-of-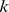 representation for the true image labels using the variable 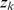, where 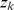 is a 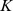-tuple with only the 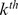 position set to one and the rest zero. For example, if the image has the label “1” (i.e., infected in this scenario), then it is represented by 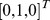, and thus 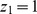 and 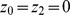. Therefore, for any image we have
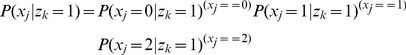
(1)where 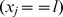 is the Boolean indicator for when user 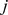 has labelled the observed image as 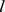. In other words, if the 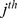 observer labels the image as “1” (i.e., infected in this scenario), then 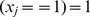 and 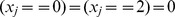. Now, we define 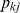 and 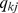 as probabilities for user 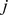 of labelling an image from the 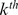 component as 0 and 1. We thus have the set of parameters shown in Table 1. The forward and decoding models of this system are shown in Figures 2 and 3, respectively.

**Table 1:** Mixture Model Parameters.

	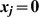	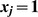	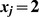
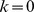	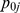	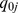	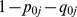
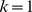	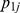	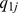	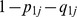
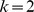	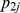	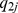	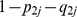

Each observation 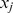 made by the 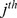 expert can take one of three category values from 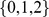. The parameters governing the model are 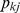 and 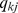, where 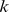 is the true category of the observed data point, and 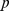 and 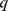 correspond to the expert labelling the data point as 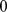 or 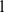, respectively.

Now suppose we have a set of 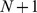 images 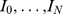, each observed and labelled by a set of 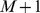 experts, with the labels represented by a matrix 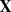 of size 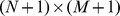. We would like to use the EM algorithm to find the correct labels 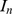. Assuming the described three component mixture model, we can write the complete data log-likelihood [32] as
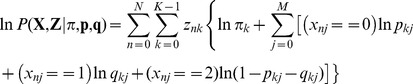
(2)where 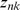 is a 1-of-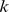 representation of the latent variables 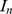 and thus 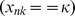 is a Boolean representing the labelling of 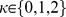 by expert 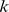 for the 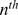 image; and 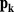 is the prior probability for the 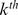 mixture component (in this case 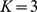). Taking the expectation with respect to the latent variables 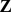 yields
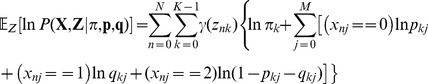
(3)where 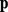 and 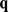 represent the set of all parameters 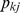 and 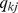 associated with the accuracy of the experts, and we have defined
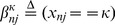
(4)and
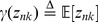
(5)

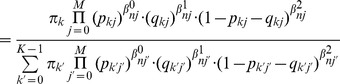


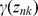
 are the “responsibilities” for component 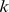 given the data point 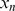 (i.e., observation vector for the nth image), which are evaluated during the “E” step of the EM algorithm. During the “M” step, we maximise the data log-likelihood with respect to the parameters 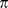, 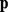 and 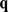. This leads to the following update equations:
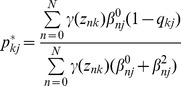
(6)

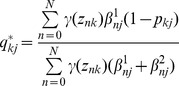
(7)

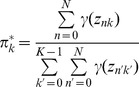
(8)


### Simulations

Since there exist no real ground-truth labels for the type of image data that we are considering in this work (i.e., microscopic images of ‘single’ RBCs that are potentially infected by malaria parasites), we will first demonstrate the viability of the EM-based algorithm through simulations. For this end, we randomly assigned labels to a simulated set of 4,000 cell images. We chose a parasitemia of 15% (i.e., 15% of the labels were 1's), a “questionable” probability of 5% (i.e., 5% of the labels were 2's), and the remaining labels (i.e., 80%) were set to 0's. Since the most difficult diagnostic task is identification of true positives, in our simulations we used more positives than typically occurring to better test the efficacy of our mathematical framework. We then simulated the responses of a set of nine experts diagnosing the images. Each individual was assigned a set of accuracy numbers (i.e., 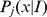) from which their responses were sampled. Once the individual responses were generated, the combined set of diagnoses was computed using Expectation Maximisation, as described above, and was compared to the original simulated cell labels, generating the combined accuracy metrics.

### From Cell-level Diagnoses to Slide-level Diagnosis

Throughout this manuscript we focus on the diagnoses of ‘single’ RBC images by experts since it is the basic task to be repeated e.g., more than 1,000 times toward accurate diagnosis of a single patient's blood smear sample. Single-cell-based analysis of a smear is essential for estimating the parasitemia, which can be quite important and valuable for monitoring the treatment of malaria patients. Often in practice however, a slide-level diagnosis is made for a patient (i.e., malaria infection observed, or malaria infection not observed). Since a thin blood smear typically contains hundreds of thousands of intact RBCs on it, slide-level malaria diagnosis using a patient's blood smear slide is relatively easier than cell-level diagnosis, as statistical errors in parasite recognition may be partially hidden. In other words, as long as the overall slide-level diagnosis is correct, the individual cell-level mistakes no longer matter (unless accurate parasitemia measurement is required for e.g., monitoring of a positive patient).

Systematic translation from the diagnoses of individual RBC images to that of a patient's blood smear is a rich topic that needs to be addressed through mathematical rigor. In the following theoretical analysis we take a detailed look at this important problem, and hypothesise that for medical professionals with different levels of expertise, the *number of RBC images that needs to be diagnosed* per blood smear sample *should vary based on their abilities*, in order to claim an accurate diagnosis per patient slide. This mathematical framework can be rather useful to customize and fine tune standard diagnostic procedures depending on the training level of the experts.

### Probabilistic framework for slide-level diagnosis from single cell diagnoses

In analysing a smear and calling it infected vs. uninfected, we can treat the formation of the slide as a stochastic process [33]. We further assume that the infected and uninfected smears follow two distinct processes with different distributions. In the case of an uninfected slide, there are no physically infected cells on the smear. Therefore, in the ideal deterministic scenario, none of the cells observed under the microscope should be labelled as infected. This however is not necessarily true, due to errors on the part of the individual (e.g., a pathologist) looking at the cells. The observer will have an error probability 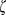 associated with her/his labels, which defines the probability of mislabelling a healthy cell as infected.

Assuming 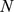 cells are observed (or labelled) from the same blood smear slide, for a healthy smear we will have the following Binomial distribution for the number of cells labelled as infected 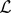


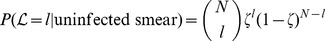
(9)The case of an infected slide (with a parasitemia rate of 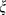), however, is much more complicated to analyse since: (1) the total number of truly infected cells (i.e., 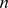) within the smear can range from 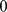 to 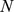 with varying probabilities; and (2) the total number of positive labels assigned to the cells by the medical expert can be due to a combination of truly infected and uninfected cells. As a result, we have the following distribution for the number of infected/positively labelled cells 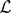 for an infected smear that has a parasitemia rate of 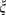:
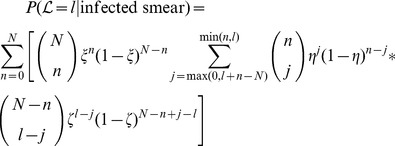
(10)where 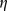 is the probability of correctly labelling an infected cell as infected.

Assuming we know the true positive and false positive probabilities, we can generate the Receiver Operating Characteristics (ROC) curves for different parasitemia levels 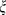 and labeled cell counts 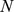.

## Results and Discussion

**Figure 4:**
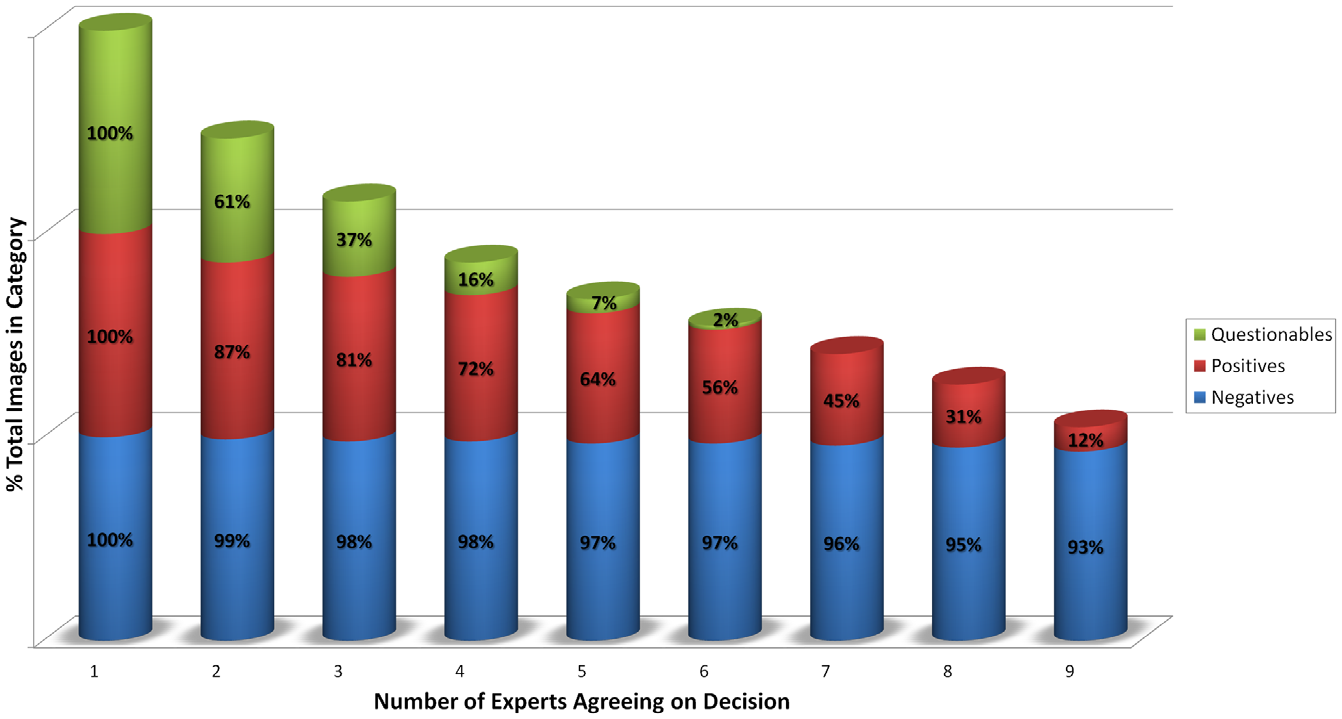
Experimental results on the level of agreement among experts. More than 8,000 RBC images were remotely presented to these experts, using the interface that is illustrated in Figure 1. For example, at least five out of nine experts agree on 97% of images labelled as negative, 64% labelled as positive, and 7% labelled as questionable; whereas only one out of nine experts at any given time agrees on the full set of labels (i.e., no two experts agree completely!). Note that these percentages are based on individual RBC images. For example, in the 3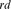 column, it is not the same 3 experts who agree on the images but possibly different sets of 3 experts for different images.

The motivation for the proposed methodology is not only to create a more accessible platform for tele-pathology, but also to increase the efficiency and accuracy of remote medical diagnosis. In other words, even relatively poorly trained medical personnel can be digitally and remotely combined to create highly accurate collective decisions (assuming each individual can perform at least better than chance in terms of accuracy). To set the stage in terms of motivation and potential severity of the problem, Figure 4 shows our experimental results, revealing the level of agreement that exists among nine highly trained medical personnel who are experts in diagnosing malaria. Given that our image database only consisted of single images of individual cells (totalling more than 8,000 RBC images) without the ability to focus in and out, we had asked these experts to label the images as infected by malaria, uninfected by malaria, or questionable (i.e., a certain judgment cannot be made). An interesting observation was the degree of variance in the expert responses as shown in Figure 4, i.e., these nine experts agreed on 93% of the images that they labelled as negative (or uninfected), and only on 12% of what they labelled as positive (or infected). Furthermore, only 64% of the images labelled as positive received that label from the majority of the experts, which implies a simple majority vote of the experts might lead to highly inefficient and potentially inaccurate diagnoses.

**Figure 5:**
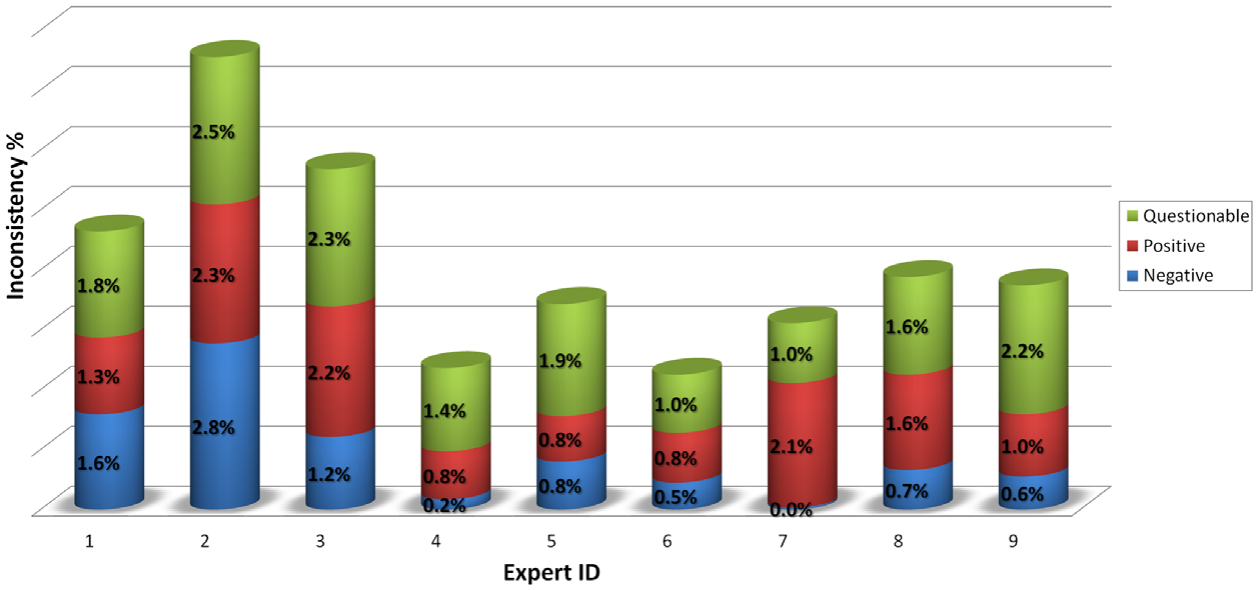
Experimental results on the level of *self-inconsistency* of each expert within each category. More than 8,000 RBC images were separately presented to these experts, using the interface that is illustrated in Figure 1. For example, expert 1 changes her/his decision regarding what s/he labelled as negative 1.6% of the time. Similarly s/he changed her/his mind for 1.3% and 1.8% of the positive and questionable images, respectively.

In addition to the inconsistencies that exist among the different experts, there is a significant amount of self-inconsistency that is exhibited by ‘each’ expert. To test the level of self-consistency of experts, each RBC image in the database was presented three times at rotations of 90 to each expert for labelling. Figure 5 shows the level of self-inconsistency that each expert exhibits within her/his responses. The most consistent expert has a self-inconsistency of 0.2% and 0.8% for the negative and positive categories, respectively, and the least consistent expert is more than 2% inconsistent in each of those categories. We can interpret this ***self-inconsistency of experts to mean that the diagnosis of an expert–even a highly trained one–is not a deterministic process***, and inherently contains a stochastic and thus random component. It also implies that ***this stochastic nature can be exploited to achieve a higher level of accuracy*** by combining diagnoses generated by multiple experts.

**Figure 6:**
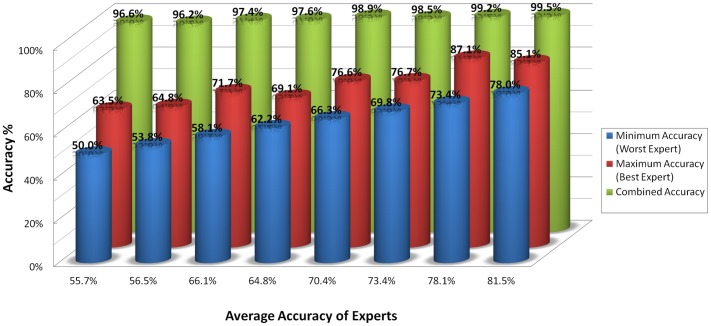
Performance results from 9 simulated experts with varying average ensemble accuracies. We can see that the combined accuracy (in green) is always higher than the maximum accuracy of the ensemble. Refer to the Methods section for details.

We must re-emphasise that for the cell images that we have used in these experiments, the true labels are not known. This is a direct consequence of the fact that we are analysing the performance of experts who would normally create such ground-truth labels. As a result, the only practical way to test the applicability and performance of our proposed methodology is to do so with simulations. Toward this goal, we created a general model of an expert's response. We assumed a model with six degrees of freedom through the parameters listed in Table 1. We ran eight simulation experiments where in each trial a pool of nine experts with varying performances were simulated (see the Methods Section). The range of overall expert accuracy for each trial was set to 10%, yielding predetermined average accuracies ranging from 55.7% to 81.5%. Running the EM-based algorithm discussed in the Methods Section on each of the simulated pools of responses, we generated the combined accuracies for these virtual experts. The results of these simulated experiments are shown in Figure 6, where we can readily observe that even when the average accuracy of the experts is less than 60%, it is possible to obtain combined accuracies that are higher than 95%. What is more interesting is the fact that the boost in accuracy that resulted from combining the multiple responses (i.e., green-coloured bars) does *not* increase at the same rate as the average accuracies of the individuals. In other words, after a certain number, subsequent addition of more experts reaches a point of diminishing returns in terms of contribution to the overall accuracy of the combined diagnosis. This can be seen as both a strength and a weakness of the proposed methodology in that if there exists a lone expert who is extremely accurate as compared to his peers within the pool, her/his responses may ‘not’ have a significant impact on the overall accuracy, and *her/his voice may get drowned by the crowd*. At the same time, a single incompetent individual cannot have a significant negative influence on the overall results. Another point that must be emphasised with regards to these simulations is that when generating the results we did not take into account the possibility that some images may be inherently more difficult to diagnose; furthermore we assumed that the errors that the experts make will be uniformly distributed across the images. Intuitively, this uniformity assumption gives each image a reasonable chance to receive more correct responses than incorrect ones. If for example, all of the experts incorrectly diagnose a set of images, then there is no way to correct those errors.

**Figure 7:**
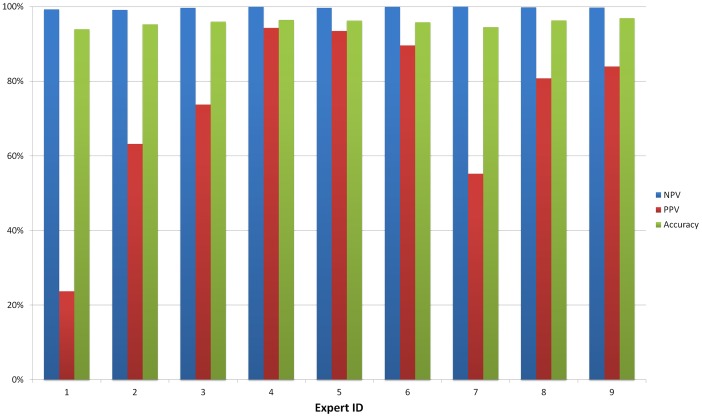
Experimental performance metrics of the experts. The metrics are calculated after combining the responses of all the experts using EM and then assuming the results to be correct. 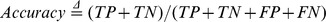, 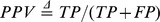, 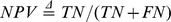, 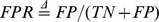, where 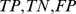, and 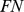 correspond to the number of true positive, true negative, false positive, and false negative labels respectively.

**Figure 8:**
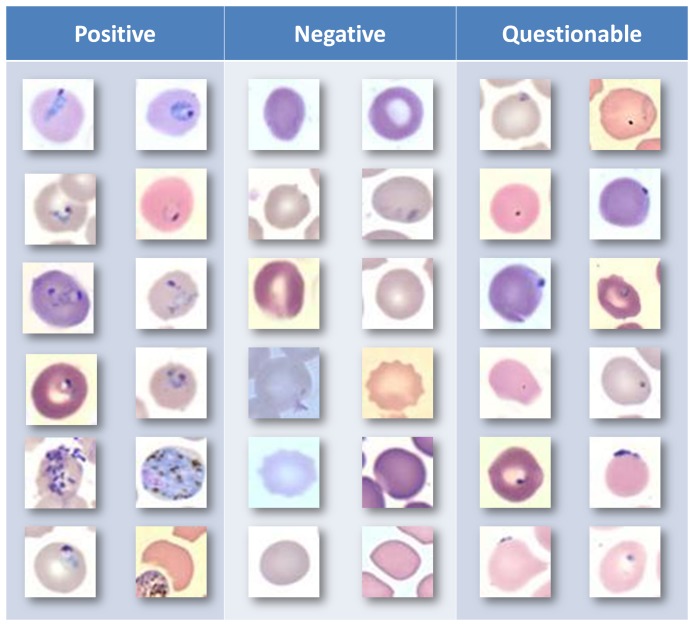
Sample cells classified by the proposed methodology. Each observation 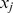 made by the 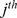 expert can take one of three category values from 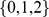. The parameters governing the model are 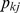 and 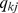, where 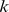 is the true category of the observed data point, and 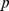 and 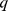 correspond to the expert labelling the data point as 0 or 1, respectively. Please refer to the Methods Section for further details.

Returning back to our experimental results with nine malaria experts, taking the EM-based consensus of the crowd to be the ground truth for the cell labels, we can generate a set of experimental performance metrics for each expert as illustrated in Figure 7. Figure 8 also illustrates some sample RBC images from the categories that resulted from this consensus. Absolute accuracy is not the best metric to measure the performance of the experts in this setting due to the significant imbalance that exists in the number of healthy and infected cells in our dataset–this imbalance is even more drastic in individual patient samples due to the low parasitemia levels that typically exist in malaria infected patients. As such, two better metrics are the *Negative Predictive Value* (NPV) and the *Positive Predictive Value* (PPV), which are indicative of the reliability of the negative and positive labels assigned to the cell images (see Figure 7). We can readily see that even though all the experts have achieved very high and similar accuracy levels, their response quality varies significantly in terms of NPV and PPV. An interesting observation can be made by comparing Figure 5 with Figure 7: experts 4, 5, and 6 who exhibited the highest levels of self-*consistency* in their responses to the uninfected and infected cell images also had the highest PPV levels.

**Figure 9:**
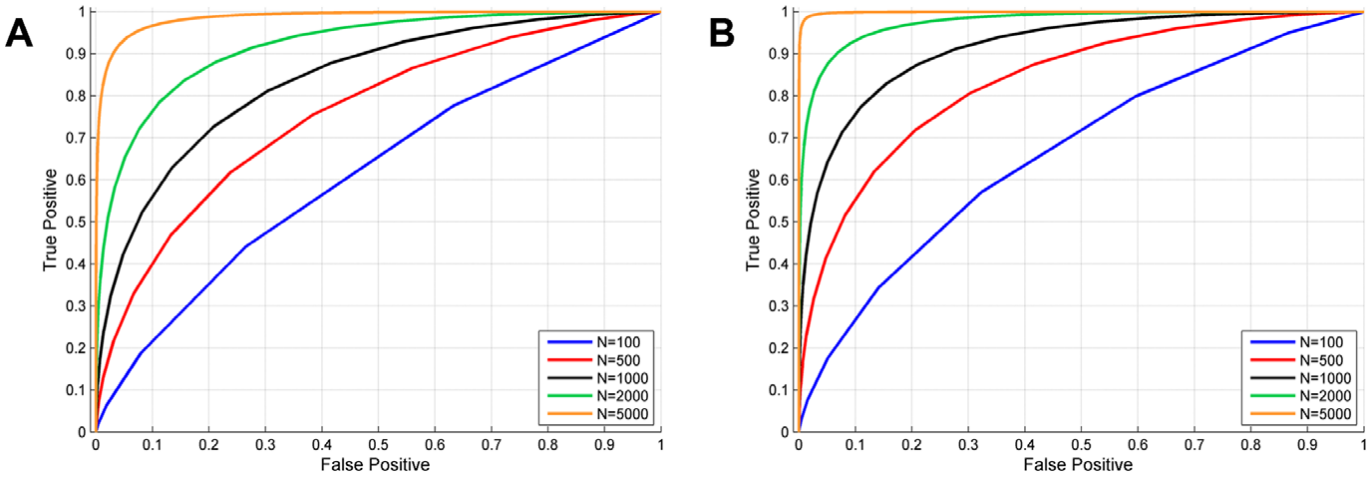
ROC curves for smear-level diagnosis. A) parasitemia 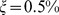. B) parasitem 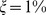.

At this point, we would like to emphasise the distinction between cell-level diagnosis and smear-level diagnosis. Although the former is a necessary step in performing the latter task, the two do not correspond to each other in a straight-forward linear fashion. As described in the Methods Section, we can use a probabilistic framework to make the transition from cell-level diagnoses to smear-level diagnosis. In doing so, we see that depending on the expertise level of the medical professional making the diagnosis, to achieve a particular level of certainty when calling a smear slide positive, with a fixed *false positive rate*, the number of individual cells that need to be examined varies drastically. For example, Figure 9 shows that when diagnosing a smear that has a parasitemia level of 0.5% (which can be typical), if the expert has a sensitivity (i.e., true positive rate) of 99%–meaning that s/he labels an infected cell correctly 99% of the time–and a false positive rate of 1% (i.e., specificity of 99%)–meaning that s/he makes the mistake of calling an uninfected cell as infected 1% of the time–s/he would then need to label more than 2,000 individual cells so that s/he would have a smear-level false positive rate less than 10% with a true positive detection rate of 80%.

We can see that the smear-level diagnosis accuracy improves as the number of labelled cells 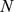 is increased. This theoretical analysis may to some extent explain the prevalence of false positive diagnoses in sub-Saharan Africa (sometimes approaching 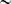
60%) [34], since even with extremely high single-cell accuracy levels, professionals can still make mistakes, and unless they observe statistically significant numbers of cells, they cannot avoid making frequent false positive diagnoses. As an example, for a parasitemia of 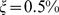, 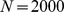, 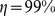, and 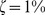, a true positive rate above 90% cannot be achieved with a false positive rate less than 30% (see Figure 9). Therefore, we believe that this mathematical framework can be generalized and used to customize and fine tune standard diagnostic procedures depending on the training levels of individual experts. Such action may lead to significant improvements in diagnosis efficiency and cost-effectiveness, especially within a digital tele-pathology platform.

We must emphasise that the cell-level and slide-level diagnosis methodologies that we have described in this manuscript were applied to thin smear samples. Under various circumstances, however, thick smear blood samples are also used for the diagnosis of malaria in the field. Though not addressed here, we believe that the multi-expert tele-diagnosis framework introduced in our work is applicable to thick smears as well. In such a scenario, there will be no cell-level diagnosis, and the thick-smear images will be cropped into smaller pieces and then sent to experts for diagnostic labelling. Instead of combining the experts' inputs to extract the infection state of individual cells, in this scenario, the experts' labels will be combined to extract the infection state of different cropped regions of the thick smear image.

At this juncture, we would like to discuss some limitations of this platform and the challenges that remain to deploy it in clinical settings. Diagnosis of malaria is inherently a binary decision (i.e., infected vs. not infected). As such, we designed and simulated the presented algorithm for the case where there are only three possible labels (including questionable) for each cell. Many medical diagnosis decisions, however, are not simple binary ones, requiring a more sophisticated multi-label decision formulation for this framework to be applicable. On the other hand, by allowing more decision possibilities in the presented algorithm, the overall performance (with the same number of experts) may also degrade. Therefore, with higher-order decisions, as there are more error possibilities, a larger crowd of experts may be necessary for generating accurate overall results. In addition to the aforementioned technical challenges that exist for the adoption of such a collaborative tele-medicine framework, there are also legal and logistics issues that need to be addressed, which is beyond the scope of this manuscript as we focus here on the technical formalism of the proposed framework.

Finally, we would like to describe our vision of how this framework may be deployed in the field. There are three necessary components for wide-scale deployment of this technology in field settings. The first is the availability of digital imaging hardware at point-of-care locations: the medical professionals and health-care workers in the field need to be able to capture digital images of a patient's blood sample (i.e., an optical microscope of sufficient quality with digital image capture capability is required). The second necessity is a reasonable Internet connection, allowing the captured images to be transmitted to servers where they would be pre-processed and distributed among medical experts. At this pre-processing stage, various computer vision and machine learning algorithms could also be applied to create a hybrid diagnosis platform as also discussed in our earlier work [30]. The final and the most crucial component in this framework is the availability of a large-enough number of experts who would agree to complete the diagnoses tasks. With these in mind, we believe that this technology will be especially valuable for remote and impoverished regions of the world where access to trained medical experts is limited. This platform significantly diminishes the need for the physical presence of the medical experts in the field, thus bringing an unprecedented level of access to medical expertise in locations and under circumstances where it was previously not possible. In this work, we believe that we have shown the viability of such a telepathology framework for efficient and accurate medical image analysis.
